# Structural alphabets for conformational analysis of nucleic acids available at dnatco.datmos.org

**DOI:** 10.1107/S2059798320009389

**Published:** 2020-08-17

**Authors:** Jiří Černý, Paulína Božíková, Michal Malý, Michal Tykač, Lada Biedermannová, Bohdan Schneider

**Affiliations:** a Laboratory of Structural Bioinformatics of Proteins, Institute of Biotechnology of the Czech Academy of Sciences, Prumyslova 595, Vestec, Czech Republic; b Laboratory of Biomolecular Recognition, Institute of Biotechnology of the Czech Academy of Sciences, Prumyslova 595, Vestec, Czech Republic

**Keywords:** structural alphabets, nucleic acids, annotation, validation, refinement

## Abstract

A detailed description of the dnatco.datmos.org web server implementing the universal structural alphabet of nucleic acids is presented. It is capable of processing any mmCIF- or PDB-formatted files containing DNA or RNA molecules; these can either be uploaded by the user or supplied as the wwPDB or PDB-REDO structural database access code. The web server performs an assignment of the nucleic acid conformations and presents the results for the intuitive annotation, validation, modeling and refinement of nucleic acids.

## Abstract 2

The dnatco.datmos.org web server performs an assignment of nucleic acid conformations and presents the results for the intuitive annotation, validation, modeling and refinement of nucleic acids.

## 1. Introduction   

In the protein world, the well known and routinely used secondary-structure elements are formally defined by local three-dimensional patterns of hydrogen-bonded amide N atoms and carbonyl O atoms of the protein backbone within a stretch of protein residues. This formal definition is often represented by a complementary description based on the occurrence of regular patterns in a series of per-residue backbone torsion angles φ and ψ. Because the protein backbone contains most of the hydrogen-bonding donor/acceptor atoms, this results in the practical equivalence of these two approaches for the description of protein secondary structure, in which a hydrogen-bonding pattern defines the backbone torsions and vice versa. Stable secondary-structure elements then also correspond to the energetically allowed regions of the two-dimensional Ramachandran plot. The per-residue description also allows the representation of proteins and their conformations as a one-dimensional string of symbols similar to the primary sequence. Depending on the level of secondary-structure description, the symbols can form variably complex structural alphabets ranging, for example, from the simplest helix–sheet–coil through the eight symbols of *DSSP* (Kabsch & Sander, 1983 ▸) and the 12 + 1 symbols of *SST* (Konagurthu *et al.*, 2012 ▸) to the 16 symbols of the Peptide Blocks (PB) alphabet (Joseph *et al.*, 2010 ▸; Craveur *et al.*, 2015 ▸), assigning a per-residue symbol to a central residue within a sliding pentapeptide window.

When defining the secondary structure of nucleic acids, the hydrogen-bonding inter­actions of pairs of nucleobases are typically considered. Because most of the hydrogen-bonding atoms in nucleic acids belong to nucleobases from sequentially mostly nonconsecutive residues and the sugar-phosphate backbone is not involved, concentrating on hydrogen bonding for the definition of local structural patterns in nucleic acids can inevitably only lead to a limited description of their true conformational variability. The nucleobase hydrogen-bonding-based description is also only possible for residues involved in base pairs and relies strongly on the accuracy and level of the annotation of hydrogen bonds between residues. It is still common that the ‘secondary structure’ of a nucleic acid is only based on the canonical Watson–Crick pairing and does not take into account the more elaborate descriptions of pairing patterns by Saenger (1984 ▸) or Leontis & Westhof (2001 ▸).

Similarly to the simplest set of protein conformations, there is a well known and comparatively simple set of nucleic acid conformations consisting of A, B and Z forms defined in the double-helical regions of nucleic acids. This oversimplified alphabet is clearly insufficient for the description of more complicated or non-double-helical structures, which include, amongst others, quadruplexes, Holliday junctions, intercalated and i-motif regions, tetraloops and a wide variety of other DNA or RNA motifs. Previous attempts to identify the complementary backbone torsion-based description of nucleic acid conformations have had to deal with two main differences when compared with the protein field. Firstly, it is not possible to assign reliable information about nucleic acid conformation to a single nucleotide; a longer stretch of residues has to be used because the atoms forming important backbone torsions formally belong to neighboring residues, and correlations between distant torsions often determine the conformation. The second and partially related difference is a result of the large number of rotatable backbone bonds that are contained in even the smallest dinucleotide building block. This leads to the necessity of working with a higher number of dimensions than in the intuitive two-dimensional φ/ψ protein-backbone torsion space. Attempts to overcome this issue and extract two-dimensional Ramachandran-like plots are based on the definition of η and θ pseudo-torsions for nucleic acids (Duarte & Pyle, 1998 ▸). This simplified level of description has been used successfully to determine and detect structural motifs within nucleic acid molecules (Humphris-Narayanan & Pyle, 2012 ▸); however, it does not capture all of the details of their conformations. An alternative approach for the description of nucleic acid conformations relies on the base parameters (Olson *et al.*, 2001 ▸) as implemented in the *DSSR* program (Lu *et al.*, 2015 ▸); however, while thoroughly implementing the description of hydrogen-bonding and stacking interactions, the base parameter-derived classification of backbone conformations into A, BI or BII forms often leads to a false conformational class. Both issues with the necessity for larger building blocks and the resulting high-dimensional data have previously been addressed by defining and using a sliding dinucleotide building block of 46 ‘suites’ for RNA (Richardson *et al.*, 2008 ▸) or 18 ‘steps’ for DNA (Svozil *et al.*, 2008 ▸) and 32 ‘steps’ for RNA (Schneider *et al.*, 2004 ▸).

**Figure 1:**
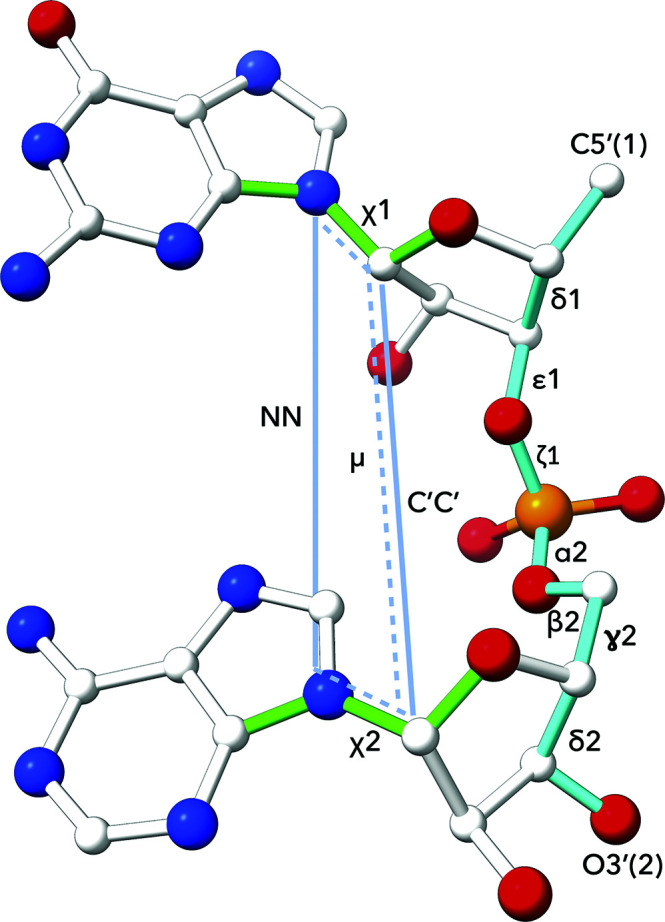
The dinucleotide step is defined by (i) seven backbone torsions, (ii) two torsions around the glycosidic bonds, (iii) one pseudo-torsion angle and (iv) two distances. The atoms involved are (i) δ1, C5′(1)—C4′(1)—C3′(1)—O3′(1); ∊1, C4′(1)—C3′(1)—O3′(1)—P(2); ζ1, C3′(1)—O3′(1)—P(2)—O5′(2); α2, O3′(1)—P(2)—O5′(2)—C5′(2); β2, P(2)—O5′(2)—C5′(2)—C4′(2); γ2, O5′(2)—C5′(2)—C4′(2)—C3′(2); δ2, C5′(2)—C4′(2)—C3′(2)—O3′(2) and (ii) χ1, O4′(1)—C1′(1)—N1/9(1)—C2/4(1); χ2, O4′(2)—C1′(2)—N1/9(2)—C2/4(2). (iii) The pseudo-torsion μ is defined as torsion between the atoms defining the glycosidic bonds of the first and second nucleotides: N1/N9(1)—C1′(1)—C1′(2)—N1/N9(2). (iv) The two distances are N1/9(1)—N1/9(2) and C1′(1)—C1′(2).

During the development leading to the currently available universal nucleic acid structural alphabet, we initially concentrated on the conformational behavior of DNA. The assignment of DNA conformations was introduced as a web server that is currently available at https://dnatco.datmos.org/v2 (Černý *et al.*, 2016 ▸). The evolved description of DNA conformations was based on a nine-dimensional space of parameters containing seven backbone torsions and two torsion angles around glycosidic bonds within a DNA dinucleotide step (Schneider *et al.*, 2018 ▸). This approach has also successfully been applied to the identification of the key structural differences between a DNA interacting with regulatory proteins and in the nucleosome core particle, explaining the structural details of different mechanisms of DNA bending (Schneider *et al.*, 2017 ▸). DNA conformations were also used for accelerated and simultaneously more accurate sampling of DNA conformations during molecular-dynamics simulations (Peter & Černý, 2018 ▸, 2019 ▸). Subsequently, we have found that for a more reliable assignment of the previously unassigned nucleic acid conformers found in intercalated and nonhelical structures, the introduction of three additional geometry parameters describing the relative orientation of bases by two additional distances and a pseudo-torsion is required. An example of a dinucleotide step with 12 parameters and the 18 atoms necessary for their calculation is shown in Fig. 1 ▸. This definition of parameters and the conformation classes derived from them are independent of the type of nucleic acid and are valid for both DNA and RNA structures.

It should be noted that although the various combinations of conformations often result in seemingly identical nucleotide morphologies, many important and biologically relevant properties of nucleic acids are conformation-dependent and differ significantly. It has been shown that backbone conformations are responsible for specific patterns of hydration (Schneider *et al.*, 1998 ▸; Biedermannová & Schneider, 2016 ▸), changes in groove widths (Schneider *et al.*, 2017 ▸) and differences in the recognition and interaction of nucleic acids and ligands (Nguyen *et al.*, 2009 ▸) or proteins (Roh *et al.*, 2009 ▸; Khesbak *et al.*, 2011 ▸; Schneider *et al.*, 2014 ▸).

## 2. Methods   

### 2.1. Derivation of the structural alphabet   

A more thorough derivation of the universal nucleic acid structural alphabet comprising of 96 conformational classes and examples of their application can be found in Černý *et al.* (2020 ▸). In brief, conformers were derived from an analysis of nearly 115 000 dinucleotide steps with an even distribution of DNA and RNA data extracted from sequentially non­redundant crystal structures with a resolution of 3.0 Å or better. Clustering in the 12D parameter space combined with empirical criteria for conformer assignment resulted in a self-consistent set of about 7000 dinucleotide steps defining the 96 conformational classes. The classification protocol then uses a *k*-nearest neighbors (k-NN) algorithm (Cover & Hart, 1967 ▸; modified by using the inverse square of periodicity-aware Euclidean distances as weights) to assign a step into one of the 96 NtC conformational classes or to the formally 97th unassigned class for conformational outliers.

### 2.2. Web server   

The web server available at https://dnatco.datmos.org is hosted as a Linux-based virtual machine in the environment provided by the ELIXIR CZ infrastructure. This ensures high availability and professional maintenance as well as easy scaling of the resources if necessary. The software part employs an Apache web server and PHP7 for the server-side scripting and JavaScript on the client side. The interactive display of analyzed 3D structures currently relies on *JSmol* (Hanson *et al.*, 2013 ▸), a JavaScript-based molecular viewer running in a browser.

The web server is internally fully mmCIF-based and an uploaded PDB- or mmCIF-formatted coordinate file is first checked for consistency using the *MAXIT* suite of programs (Feng, 2017 ▸). If necessary, data categories are constructed, residue numbering and atom names are modified according to standards and the input file is then converted to the mmCIF format. The mmCIF file is then processed using an in-house Python script that extracts torsional parameters in the detected dinucleotide steps. Only steps composed of standard or modified residues containing all 18 atoms can be assigned by the underlying modified k-NN algorithm.

## 3. Results and discussion   

The current universal nucleic acid structural alphabet comprising of 96 + 1 (di)nucleotide conformer (NtC) conformational classes is a significant extension of the simple A/B/Z classification and, compared with the typically base-stacked conformers of A, B and Z character, can also describe a wide range of steps with bases that are unstacked and more distant, belonging to intercalated or open conformations. For easier analysis, these 96 conformer classes can be also grouped by similarity to form a more compact structural alphabet called CANA (Conformational Alphabet of Nucleic Acids) that contains 14 + 1 symbols.

Our recent analysis of over 11 000 DNA or RNA structures available in the wwPDB, containing over 5.8 million steps, revealed 30% of analyzed steps to be unassigned conformational outliers. However, a significant proportion of them are close to some of the known NtC classes. These unassigned dinucleotides represent incompletely refined portions of the nucleic acid structures and we believe that with the help of proper refinement tools they can be re-refined into the corresponding conformational class.

**Figure 2:**
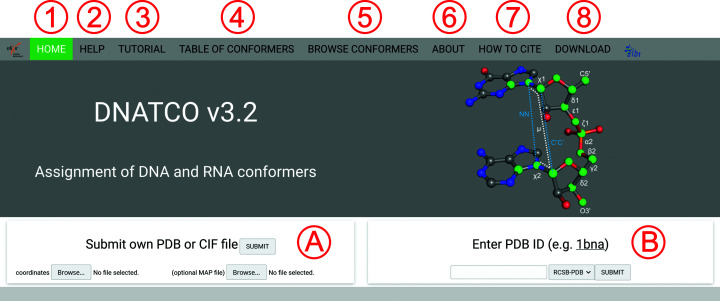
Snapshot of the Front page showing the tabs (labeled **1**–**8**) at the top of the page as described in more detail in Section 3.1. The middle part shows the definition of a dinucleotide step with 12 parameters (white text for torsions and blue for distances) and the 18 atoms (green spheres) necessary for their calculation. The bottom part of the page allows the upload of user-provided coordinates (**A**) or the analysis of database-deposited structures (**B**).

**Figure 3:**
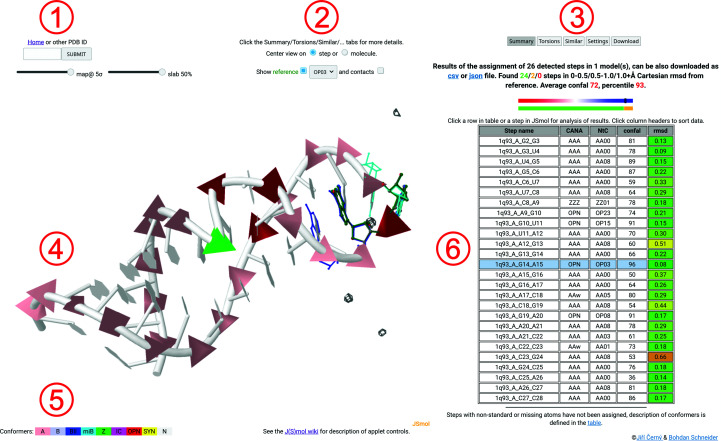
Snapshot of the Results page showing a typical representation of the conformations assigned to a nucleic acid structure. The sarcin/ricin loop structure with PDB code 1q93 (Correll *et al.*, 2003 ▸) is used as an example. The figure demonstrates the intuitive annotation and simple recognition of structural features and motifs in the structure. The regions labeled **1**–**6** are described in more detail in Section 3.2.

The dnatco.datmos.org web server implementing the annotation, validation, modeling and refinement of nucleic acid structures employing the NtC-based structural alphabet of nucleic acids is organized into two main pages: the Front page, shown in Fig. 2 ▸, and the Results page, shown in Fig. 3 ▸.

### 3.1. The Front page   

As shown in Fig. 2, the top part of the Front page collects eight tabs for simple access to the assignment of DNA and RNA conformers as well as to additional information about the web server, the underlying definition of conformers with their description as a table and downloadable files, and access to a database of assignment results. The Home tab (labeled **1** in Fig. 2 ▸) provides the definition of a dinucleotide step with 12 parameters (white text for torsions and blue for distances) and the 18 atoms (green balls) necessary for their calculation. The bottom part of the page allows the upload of user-provided mmCIF- or PDB-formatted coordinate files with an optional CCP4/MRC electron-density map (labeled **A** in Fig. 2 ▸). Analysis of coordinates deposited in the wwPDB (Berman, Battistuz *et al.*, 2002 ▸; Burley *et al.*, 2019 ▸) and PDB-REDO (Joosten *et al.*, 2012 ▸, 2014 ▸) structural databases is available from the right-hand side of the page (labeled **B** in Fig. 2 ▸). After pressing the SUBMIT button, the converted or deposited mmCIF file is then processed, the conformation of the dinucleotide steps is assigned and the results are displayed.

The Help section (labeled **2**) in Fig. 2 ▸ mainly contains a short summary of the assignment protocol and the definition of nucleic acid conformers at the nearly dinucleotide-step level. The set of representative conformer geometries extracted from crystal structures is available for download. The section also explains the general principles of the four-letter conformer nomenclature using the system combining the A, B, Z, IC (intercalated), OP (open) and S (for steps with a base in a *syn* orientation) symbols with a numeric code. Further, a gallery of supported residues is provided, displaying known residues containing the 18 expected standard atom names. The gallery summarizes over 300 residues and provides a graphical reference as well as links to the PDB.

The Tutorial section (labeled **3**) in Fig. 2 ▸ shows an example of conformer assignment using the B-DNA dodecamer structure in PDB entry 1bna (Drew *et al.*, 1981 ▸). The content of this publication will later serve as a tutorial, possibly supplemented by a video tutorial.

The Table of Conformers section (labeled **4**) in Fig. 2 ▸ provides a detailed description of the 96 known nucleic acid conformers, showing their annotation, their corresponding CANA and NtC codes and the frequency of their occurrence in the analyzed sequentially nonredundant set of high-resolution crystal structures, as well as the average values of the 12 sugar-phosphate backbone parameters defining each conformation.

The Browse Conformers section (labeled **5**) in Fig. 2 ▸ provides searchable access to the database of occurrences of all 96 NtC conformational classes across the PDB structural database. The query results are summarized in an interactive table, allowing a deeper analysis of the selected step.

The About section (labeled **6**) in Fig. 2 ▸ shows a log of the evolution of the dnatco.datmos.org web server, with links to access previous versions and a short summary of the main features of each publicly released version.

The How to Cite section (labeled **7**) in Fig. 2 ▸ provides previous publications describing the web server and the selection, definition and an example of the application of the nucleic acid structural alphabet.

The Download section (labeled **8**) in Fig. 2 ▸ offers the download of files containing the definitions of conformers as average values of their geometrical parameters (in CSV format) with corresponding e.s.d. values (in CSV format), and a ZIP archive of PDB-formatted files with Cartesian coordinates of representative examples for each conformer.

### 3.2. The Results page   

Fig. 3 ▸ demonstrates the intuitive annotation and simple recognition of structural features and motifs in the example sarcin/ricin loop structure with PDB code 1q93 (Correll *et al.*, 2003 ▸). While the pink-colored pyramids represent ‘standard’ A forms occurring typically in (double) helices, there are four steps colored in red and one in green highlighting steps in the open and Z forms, respectively. Indeed, these regions correspond to the bulged-G motif represented by the sequence of NtC symbols AAxx-ZZ01-OP23-OP15-AAxx and interacting across the strand with the OP08 step. The AAxx-OP03-AAxx sequence of NtC symbols is further characteristic (Černý *et al.*, 2020 ▸) of the GNRA tetraloop (Woese *et al.*, 1990 ▸; Heus & Pardi, 1991 ▸).

As shown in Fig. 3 ▸, the main regions of the Results page contain electron-density map sigma and slab-value controls located below the quick assignment input box in the region labeled **1** in Fig. 3 ▸. Controls in the region labeled **2** in Fig. 3 ▸ allow the reference frame to be changed, the view to be centered on the step or on the whole structure, the reference steps to be shown, the NtC class of the displayed reference to be changed and the atomic coordinates of residues in contact with the selected step to be displayed. If the analyzed structure contains multiple models and/or alternate positions, the region also shows additional related controls. The upper part of the region labeled **3** in Fig. 3 ▸ contains buttons that switch to various levels of information. The lower part usually shows the title of the analyzed structure, a link to the PDB web page for the structure and a summary of the assignment including the number of steps in three Cartesian r.m.s.d. ranges, the total confal quality score and a percentile for comparison with other structures. These values are also shown in a graphical form below. The full CSV-formatted report can be downloaded using the provided link. The region labeled **4** in Fig. 3 ▸ shows a *JSmol*-based 3D visualization of the nucleic acid. If selected in the region labeled **2**, the applet also shows a structure superposition of the reference NtC using representative structures for the selected step (green) and for the previous (blue) and next (cyan) overlapping steps in the structure. The graphical representation allows intuitive visual annotation and validation of the structure. Color-coded phosphate-centered pyramids carry information about the group of NtC classes that each step belongs to (see the region labeled **5** in Fig. 3 ▸ for color definitions). Confal scores for each step are also encoded by the size of the pyramid, with higher scores and a larger pyramid corresponding to a better match to the NtC class. The region labeled **6** in Fig. 3 ▸ contains a table summarizing the assignment for each detected step. The columns in the table contain step names, CANA and NtC alphabet codes, confal values and the color-coded Cartesian r.m.s.d. values for each step compared with the corresponding NtC reference. Each column can be sorted by clicking the column headers. Hovering over a table row shows a short annotation for the step. Clicking a table row or a step in the *JSmol* applet highlights the step in the table as well as in the applet and updates therelated parts of the page. The step name in the table has the general form PDBID[-mmodel#]_chain_resname[.altloc]resnr[.inscode]_resname[.altloc]resnr[.inscode], while an extra underscore between each ‘resname’ and ‘resnr’ is used internally.

### 3.3. Analysis of the results for OP03 step 1q93_A_G14_A15   

**Figure 4:**
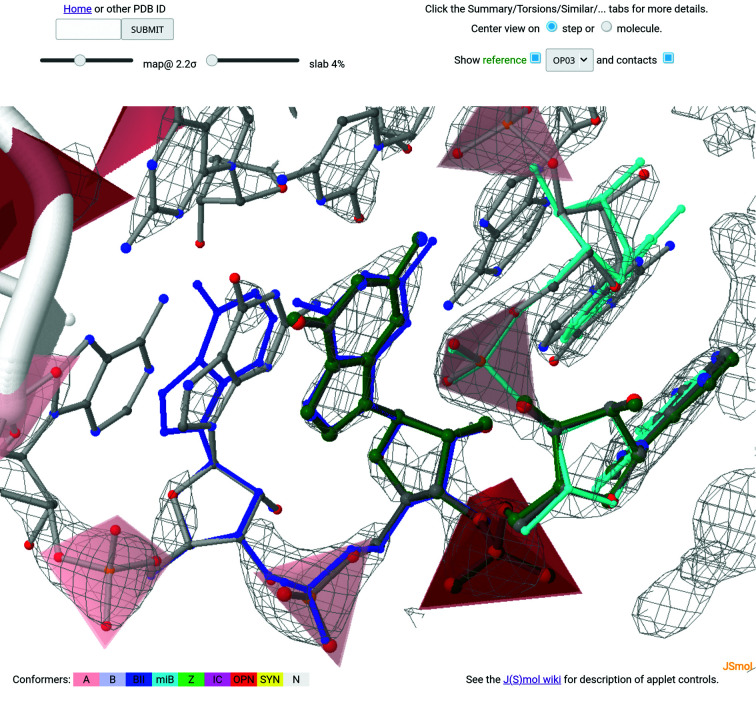
Enlargement of the selected 1q93_A_G14_A15 step, showing the details of the overlapping reference steps AA00 (blue sticks) for G13_G14, OP03 (green sticks) for G14_A15 and AA00 (cyan sticks) for A15_G16. With the ‘contacts’ checkbox active, the residues and atoms around the selected step are shown in gray. The density-map sigma as well as the slab-control values are set for clarity.

**Figure 5:**
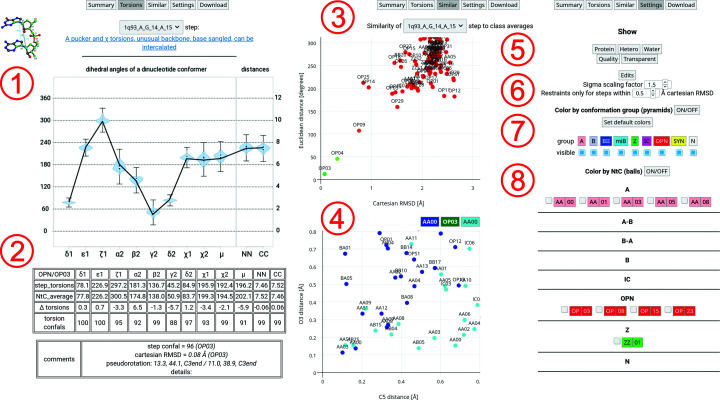
A collage of detailed results for the 1q93_A_G14_A15 step. The regions labeled **1**–**8** are described in more detail in Section 3.3.

As shown in Fig. 3 ▸, the 1q93_A_G14_A15 step is assigned to the OP03 NtC class, which is a representative of the open conformations, having an A-like sugar pucker and χ torsions, an unusual backbone and bases that are angled (not coplanar). The confal score of the step is 96, indicating very high similarity to the average values that define the conformation. A very low Cartesian r.m.s.d of 0.08 Å is calculated for the 18 atoms defining the step compared with the best representative of the OP03 class. Fig. 4 ▸ then shows an enlargement of the selected 1q93_A_G14_A15 step with details of the overlapping reference steps AA00 (blue sticks) for G13_G14, OP03 (green sticks) for G14_A15 and AA00 (cyan sticks) for A15_G16. A visual inspection of the overlapping steps indicates relatively good compatibility of the steps. The ‘contacts’ checkbox is active in Fig. 4 ▸, leading to the visualization of residues and atoms around the selected step as gray sticks. A series of more quantitative measures can be accessed by clicking the buttons above the table (shown in region **3** of Fig. 3 ▸). A summary of these details is provided as a collage in Fig. 5 ▸.

The Torsions tab in region **1** of Fig. 5 ▸ shows a plot of the values of NtC parameters displayed as a black line on top of a violin plot showing the distribution of parameters in experimental structures and ‘error bars’ indicating the lowest and highest allowed displacement from the mean parameter value. The ‘error bars’ for each parameter also define the border values for the confal function. The confal function is a Gaussian function defined in such a way that it reaches a value of 100 at the average value of the parameter and a value of 1 at the border closer to the average. The confal value is set to 0 for more distant values. The confal score for a step is then calculated as a harmonic mean of its 12 confal values, and the confal score for a structure is calculated as an average of the step values. Region **2** in Fig. 5 ▸ shows a table with detailed differences and confal values for each parameter within a step. When a different NtC is chosen for the reference superposition, the plot and table are updated, with changes indicated in red text.

The Similar tab in the middle of Fig. 5 ▸ shows two interactive scatter plots. The plot in region **3** of Fig. 5 ▸ summarizes for the selected step the correlation of the Cartesian r.m.s.d. values for all 96 NtC class references with their Euclidean distances. Points in the plot are color-coded from green for Cartesian r.m.s.d. values below 0.5 Å through yellow to red for Cartesian r.m.s.d. values over 1.0 Å. For the selected 1q93_A_G14_A15 step in the assigned OP03 conformation the ‘similarity plot’ shows that it is also structurally similar to the OP04 class, while other conformations are distant both in Euclidean and in Cartesian space. Clicking an NtC named point changes the reference for the superposition of the active step in the *JSmol* applet. The plot in region **4** of Fig. 5 ▸ shows the ‘connectivity plot’, summarizing distances in ångströms between overlapping C5′ and O3′ atoms of the previous (blue points) and next (cyan points) steps. The plot shows that the previous G13_G14 AA00 step overlaps very well, while the C5′ distance of the next A15_G16 AA00 step is about 0.68 Å. The similarity plot for the A15_G16 step suggests the AA08 class as an alternative. AA08 would be a similarly well fitting conformation, with better connectivity to the A15_G16 step. Although the resolution of PDB entry 1q93 does not allow an electron-density-guided choice, the high-resolution (1.04 Å) structure PDB entry 1q9a (Correll *et al.*, 2003 ▸) contains the corresponding step in the AA08 conformation. The AA08 conformation is also present in the PDB-REDO re-refined 1q93 structure.

The Settings tab on the right-hand side of Fig. 5 ▸ shows various controls for visual representation of the structure. The region **5** in Fig. 5 ▸ contains buttons to toggle the visibility of protein residues, nonprotein/nucleic acid residues and water molecules. The second row toggles higher quality rendering in *JSmol* and the transparency of pyramid and sphere objects. Region **6** in Fig. 5 ▸ controls the parameters and visibility of restraint-related functionality. The restraint scaling factor and the Cartesian r.m.s.d. cutoff can be adjusted. The active ‘edits’ mode also shows an additional column in the table on the Summary tab. Region **7** in Fig. 5 ▸ controls the visibility and colors of pyramids representing NtC groups. Region **8** in Fig. 5 ▸ allows the display and color setting of the alternative graphical representation of nucleic acid structures. The user-adjustable color-coded visualization allows simple highlighting for each conformer. The choice of C5′ and O3′ atoms for the backbone representation allows an intuitive detection of shape irregularities in the structure. See Supplementary Fig. S1 for an example of the representation.

### 3.4. Analysis of results for AA08 step 1q93_A_C18_G19   

**Figure 6:**
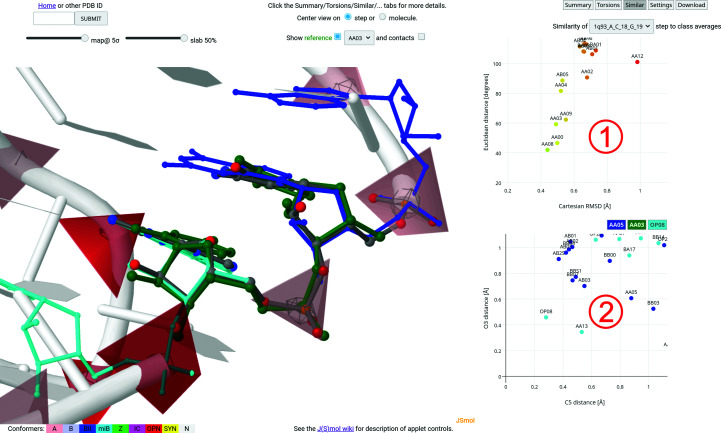
Analysis of similarity (**1**) and connectivity (**2**) plots for the 1q93_A_C18_G19 step. The similarity plot shows a relatively common case in which a range of NtC conformers, AA08, AA00, AA03, AA09, AA04, AB05 and so on, share a similar overall 3D shape as given by the Cartesian r.m.s.d. value. These cases in general show the strength of the backbone torsion-based assignment process in distinguishing the most probable conformational class of the step from the set of populated clusters.

**Figure 7:**
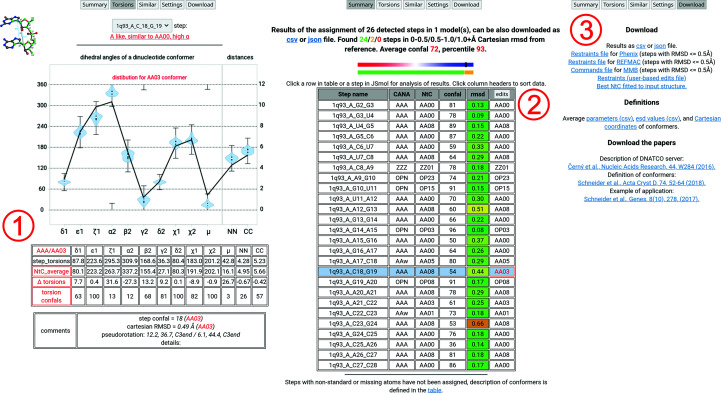
A collage of detailed results for the 1q93_A_C18_G19 step. The regions labeled **1**–**3** are described in more detail in Section 3.4.

The next, more complicated example will analyze the 1q93_A_C18_G19 step conformation. As shown in Fig. 6 ▸, similarity (region **1**) and connectivity (region **2**) plots for the C18_G19 step demonstrate a relatively common case in which a range of NtC conformers, in this case AA08, AA00, AA03, AA09, AA04, AB05 and so on, share a similar overall 3D shape as given by the Cartesian r.m.s.d. value. These cases in general show the strength of the backbone torsion-based assignment process in distinguishing the most probable conformational class of the step based on the set of populated clusters. Fig. 7 ▸ summarizes the details of the C18_G19 step after changing the conformation to the AA03 class selected on the basis of smaller connectivity-plot values. The Torsions tab in region **1** of Fig. 7 ▸ summarizes AA03 class differences, which are highlighted in red. The ζ1, α2 and μ parameters differ most from the current step geometry; however, for demonstration purposes we could follow this direction. The Summary tab in region (**2**) of Fig. 7 ▸ shows the additional column that is uncovered by clicking the edits button in the Settings tab. The table now contains a single modification from AA08 to AA03 for the C18_G19 step, which is highlighted in red. Clicking the edits button in the header of the additional column generates a range of files available for download from the Download tab, shown as region **3** in Fig. 7 ▸ next to the previously mentioned definitions of conformers and the relevant papers. In the upper part of the region there are restraint files available for *phenix.refine* (Afonine *et al.*, 2012 ▸), *REFMAC* (Nicholls *et al.*, 2012 ▸) and *MMB* (Flores & Altman, 2010 ▸). A more user-friendly implementation of NtC-based restraints into these programs is under development; however, we have already successfully used a semi-automatic procedure for the refinement of three new crystal structures (Kolenko *et al.*, 2020 ▸). Furthermore, an mmCIF-formatted coordinate file containing superposed representative conformers for each identified step in the analyzed structure is also provided.

## 4. Conclusions   

The dnatco.datmos.org web server provides tools for the intuitive annotation and validation of nucleic acid structures employing the NtC-based structural alphabet of nucleic acids. The results of the assignment are available as CSV-formatted files as well as in JSON format. Alternatively, we will produce enhanced mmCIF files containing the NtC-related extension of the mmCIF format developed in collaboration with the RCSB team. The dnatco.datmos.org web server is also accessed by the Nucleic Acid Database (NDB; Berman, Westbrook *et al.*, 2002 ▸) as one of its suggested validation tools. The web server also provides user-adjustable restraint files for the refinement and modeling of nucleic acid structures. A set of Python scripts for programmatic access to the web server is available to the interested users from the Download tab of the Front page.

## Supplementary Material

Supplementary Figure S1. DOI: 10.1107/S2059798320009389/ir5007sup1.pdf
