# Supplementary material for: Structural alphabets for conformational analysis of nucleic acids available at dnatco.datmos.org
Source: Acta Crystallogr D Struct Biol. 2020 Aug 17;76(Pt 9):805–13. doi: 10.1107/S2059798320009389 (PMC7466747; doi:10.1107/S2059798320009389)
Supplement: Supplementary file 1 [file d-76-00805-sup1.pdf]

Acta Cryst D
STRUCTURAL BIOLOGY

Volume 76 (2020)

Supporting information for article:

Structural alphabets for conformational analysis of nucleic acids available at dnatco.org

Jiří Černý, Paulina Božíková, Michal Malý, Michal Tykač, Lada Biedermannová and Bohdan Schneider

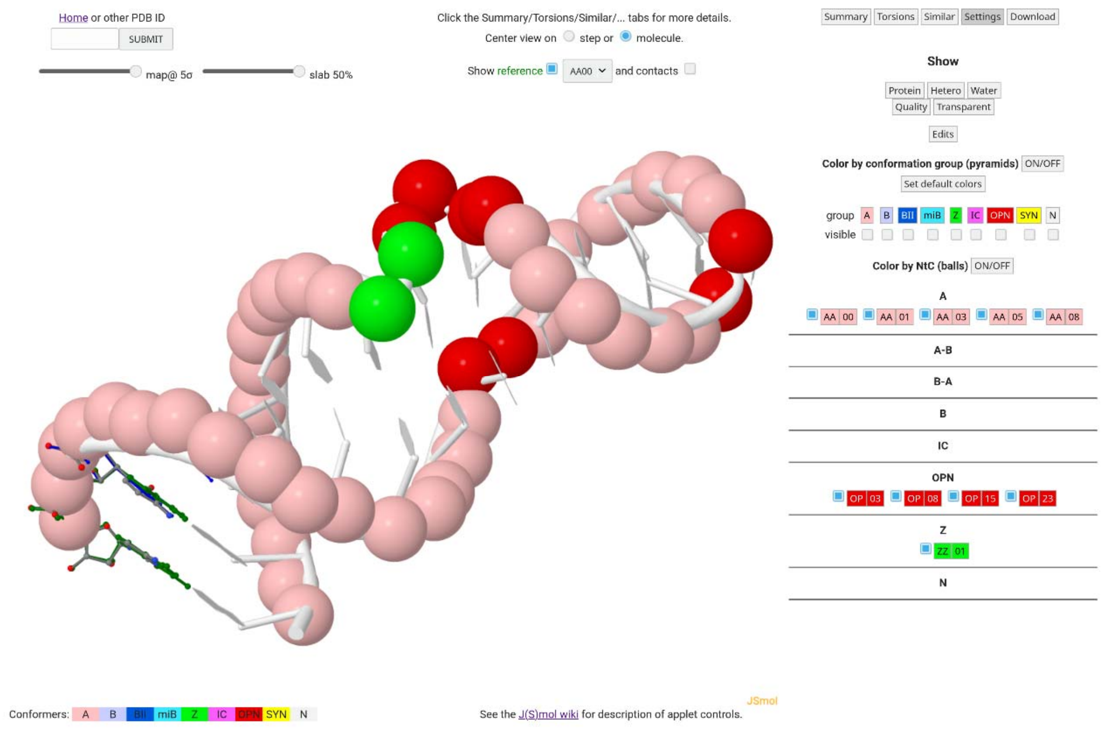

Figure S1 Snapshot of the alternative graphical representation of nucleic acid structures. The user adjustable color coded visualization allows simple highlighting for each conformer. The choice of C5' and O3' atoms for the backbone representation allows an intuitive detection of shape irregularities in the structure.
